# Incorporation of Bismuth(III) Oxide Nanoparticles into Carbon Ceramic Composite: Electrode Material with Improved Electroanalytical Performance in 4-Chloro-3-Methylphenol Determination

**DOI:** 10.3390/ma17030665

**Published:** 2024-01-29

**Authors:** Mariola Brycht, Andrzej Leniart, Sławomira Skrzypek, Barbara Burnat

**Affiliations:** University of Lodz, Faculty of Chemistry, Department of Inorganic and Analytical Chemistry, Tamka 12, 91-403 Lodz, Poland; mariola.brycht@chemia.uni.lodz.pl (M.B.); andrzej.leniart@chemia.uni.lodz.pl (A.L.); slawomira.skrzypek@chemia.uni.lodz.pl (S.S.)

**Keywords:** bulk modification, electrochemical characterization, surface characterization, effective surface area, pollutant determination

## Abstract

In this study, a carbon ceramic electrode (CCE) with improved electroanalytical performance was developed by bulk-modifying it with bismuth(III) oxide nanoparticles (Bi-CCE). Characterization of the Bi-CCE was conducted employing atomic force microscopy, scanning electron microscopy with energy-dispersive X-ray spectroscopy, cyclic voltammetry (CV), and electrochemical impedance spectroscopy. Comparative analysis was conducted using an unmodified CCE. The findings proved that the incorporation of Bi_2_O_3_ nanoparticles into the CCE significantly altered the morphology and topography of the ceramic composite, and it improved the electrochemical properties of CCE. Notably, the Bi-CCE demonstrated a prolonged operational lifespan of at least three months, and there was a high reproducibility of the electrode preparation procedure. The developed Bi-CCE was effectively employed to explore the electrochemical behavior and quantify the priority environmental pollutant 4-chloro-3-methylphenol (PCMC) using CV and square-wave voltammetry (SWV), respectively. Notably, the developed SWV procedure utilizing Bi-CCE exhibited significantly enhanced sensitivity (0.115 µA L mol^−1^), an extended linearity (0.5–58.0 µmol L^−1^), and a lower limit of detection (0.17 µmol L^−1^) in comparison with the unmodified electrode. Furthermore, the Bi-CCE was utilized effectively for the detection of PCMC in a river water sample intentionally spiked with the compound. The selectivity toward PCMC determination was also successfully assessed.

## 1. Introduction

Over the past several years, significant attention has been devoted to advancing a diverse range of carbon-based electrode materials. These electrodes offer numerous advantages, including straightforward preparation, extended durability, a wide potential window, and facile surface renewability [1]. Carbon-based electrodes find extensive use in electrochemical sensors, facilitating the identification of diverse substances such as pollutants, biomolecules, and drugs [1,2,3,4]. Additionally, carbon-based materials are crucial in biosensors, contributing significantly to the detection of biological molecules. This makes them invaluable in applications such as medical diagnostics, environmental monitoring, and ensuring food safety [2,3,5,6,7]. Furthermore, these electrodes are utilized in electroanalytical techniques, aiding in the precise determination of trace elements and the exploration of reaction mechanisms [8,9].

Among the spectrum of carbon-based electrodes, carbon ceramic electrodes (CCEs) pioneered by Tsionsky et al. in 1994 [10] occupy a prominent position. Fabricated through a sol–gel approach integrating carbon powder (graphite) into a silica sol–gel matrix [11], CCEs not only showcase the above-mentioned features of carbon-based electrodes but also exhibit exceptional mechanical resistance and robustness [12]. The versatility of CCEs for modification through diverse techniques stands as a crucial advantage, enhancing their electroanalytical capabilities. Surface modifications of CCEs achieved via electrodeposition [13,14,15] or drop-casting procedure [16,17,18] contribute to improved electrode sensitivity. However, these approaches pose challenges related to surface renewal, demanding the preparation of new modification layers and potentially leading to inconsistent results. Moreover, these modification techniques encounter limitations in controlling electrode surface film thickness [12]. An efficient alternative to the surface modification procedure, ensuring greater result reproducibility, involves the bulk modification of the electrode material. In the case of CCEs, this approach includes the partial [19,20,21,22,23] or complete [24,25,26,27] replacement of the original carbon material (graphite) in the silicon matrix with other carbon-based materials, such as carbon nanotubes [19,24,25,26,27], graphene oxide [20], carbon black [21], and non-carbon materials like nanoparticles [23,28], zeolites [22], and Prussian blue [29]. The advantage of this approach lies in obtaining a carbon–ceramic composite with an evenly distributed modifier throughout the entire volume of the electrode material. This allows for straightforward surface renewal through mechanical polishing while maintaining surface reproducibility after each polishing step [12].

Metal oxide nanoparticles have gathered significant attention in the electrochemistry due to their unique properties, making them ideal for fabricating electrochemical sensors used in electroanalysis [30]. Bismuth(III) oxide (Bi_2_O_3_) nanoparticles (Bi_2_O_3_NPs) exhibit promising electronic characteristics, including a low-energy bandgap, large surface area, electrochemical stability, and catalytic behavior, making them well-suited for various applications [30,31,32]. Although Bi_2_O_3_NPs have primarily functioned as surface modifiers for glassy carbon electrodes and screen-printed electrodes, their utilization as a bulk modifier has been limited to carbon paste electrodes. It is envisioned that employing Bi_2_O_3_NPs as a bulk modifier of CCEs will result in electrode materials with significantly enhanced surface properties, an enlarged electroactive surface area, and improved electroanalytical performance.

Phenolic compounds have been designated as high-priority pollutants by both the United States Environmental Protection Agency and the European Union due to their known toxicity. These chemicals exhibit significant short- and long-term adverse effects on both human health and animal well-being. The existence of phenolic compounds in the aquatic environments is not only undesirable but also poses a significant threat to human health and wildlife. Consequently, various wastewater treatment methods have been developed and implemented to eliminate phenolic compounds from industrial, domestic, and municipal wastewater streams before their release into water bodies [33]. Moreover, numerous procedures based on chromatographic and electroanalytical methods have been developed for the quantitative determination of phenolic compounds in water. One notable phenolic priority environmental pollutant is 4-chloro-3-methylphenol (synonym: 4-chloro-m-cresol, PCMC), which is used as a medicinal or non-medicinal ingredient in final pharmaceuticals, disinfectants, veterinary drug products, and cosmetics, and it is used as an active ingredient in registered pest control products. Due to the wide use of PCMC, it is necessary to monitor its content in the aquatic environment to prevent its destructive effects on both human and aquatic lives. In the literature, only a few reports on electroanalytical procedures for PCMC determination using carbon-based electrodes can be found [24,34,35,36,37,38]. One of these reports is from our previous study, in which a voltammetric procedure for PCMC determination involving CCE modified with carbon nanotubes was detailed [24]. In that study, a linear range of 3–32 µmol L^−1^ and a limit of detection (LOD) of 0.71 µmol L^−1^ was achieved. Considering the potential for improvement, we hypothesize that using Bi_2_O_3_NPs to prepare a modified CCE could enhance the performance of such a voltammetric procedure. To our knowledge, this specific electrode type has not been employed for PCMC determination previously.

Our study aims to prepare a bulk-modified CCE by incorporating Bi_2_O_3_NPs (Bi-CCE), replacing a portion of graphite within a ceramic composite. The investigation focuses on assessing how the incorporation of Bi_2_O_3_NPs influences the surface characteristics, electrochemical properties toward the ferro/ferricyanide ([Fe(CN)_6_]^4−^/[Fe(CN)_6_]^3−^) redox model system, and the overall electroanalytical performance of the Bi-CCE toward the priority environmental pollutant PCMC.

## 2. Materials and Methods

### 2.1. Materials

For the CCEs preparation, methyltrimethoxysilane (MTMS, 98%, Sigma Aldrich, Warsaw, Poland), methanol (CH_3_OH, 99.8%, Avantor Performance, Gliwice, Poland), and hydrochloric acid (HCl, pure p.a., 36–38%, Avantor Performance, Gliwice, Poland) were employed without undergoing any further purification. Graphite flakes (99%, 7–10 micron, Alfa Aesar, Karlsruhe, Germany) served as the carbon material for the CCEs. Bi_2_O_3_NPs (99.999%, particle size of 90–210 nm, Sigma Aldrich, Warsaw, Poland) were utilized as the modifier in the Bi-CCE preparation.

All chemicals employed were of analytical reagent grade, and the solutions were prepared using triply distilled water. The electrochemical characterization of the CCEs involved the use of 1.0 mol L^−1^ potassium chloride (KCl, 99%, Avantor Performance, Gliwice, Poland) solution and 1.0 mmol L^−1^ potassium ferricyanide (K_3_[Fe(CN)_6_], 99%, Avantor Performance, Gliwice, Poland) solution. For the preparation of a 1.0 mmol L^−1^ PCMC stock solution, PCMC (99%, Sigma Aldrich, Warsaw, Poland) was dissolved in water and stored in a glass flask in a refrigerator when not in use. For the preparation of the Britton–Robinson buffer (BRB) across a pH range of 2.0–12.0, the following reagents were utilized: phosphoric acid (H_3_PO_4,_ 85.0%, Avantor Performance, Gliwice, Poland), boric acid (H_3_BO_3,_ pure p.a., Avantor Performance, Gliwice, Poland), and acetic acid (CH_3_COOH, pure p.a., 99.5%, Avantor Performance, Gliwice, Poland), all at a concentration of 40.0 mmol L^−1^. Additionally, sodium hydroxide (NaOH, pure p.a., Avantor Performance, Gliwice, Poland) at a concentration of 0.20 mol L^−1^ was employed to adjust the pH values of the buffer solutions. River water samples, collected from the Rudawa River at coordinates 50.057040, 19.906389, were utilized for the analytical purposes. These samples, spiked with the known concentration of PCMC, underwent examination without any pretreatment or filtration except for dilution. The samples were stored in a refrigerator before experiments and analyzed within one week of collection. The concentration of stock solutions of interferents (Cd^2+^, Ni^2+^, Cu^2+^, HCO_3_^−^, and SO_4_^2−^; all from Avantor Performance, Gliwice, Poland) was 1.0 mmol L^−1^.

### 2.2. Preparation of the CCEs

To prepare Bi-CCE, the sol–gel method was employed. Initially, a mixture containing 750 µL of CH_3_OH as the solvent, 500 µL of MTMS serving as the silica matrix precursor, and 50 µL of concentrated HCl as the catalyst was stirred for 5 min using a magnetic stirrer set at 450 rpm. Next, 600 mg of the activated graphite (prepared following the procedure outlined in [39]) was combined with 150 mg of Bi_2_O_3_NPs in the silica sol solution. This mixture was thoroughly stirred using a spatula and then promptly transferred into a Teflon tube (measuring 5 mm in length and 3 mm in inner diameter), and silver-painted copper wire (1 mm in diameter) was used as the electrical contact. The unmodified CCE was prepared analogously with the only difference being the addition of 750 mg of activated graphite into the silica sol solution. The resulting CCEs were then air-dried for 48 h at room temperature. The CCEs were polished using 2000 grit polishing paper, which was followed by a cleansing with water and drying using argon. This process was performed before their initial use and repeated before each measurement series.

### 2.3. Apparatus

An atomic force microscope (AFM, Dimension Icon, Bruker Corporation, Santa Barbara, CA, USA) and field emission scanning electron microscope (SEM, Nova NanoSEM 450, FEI, USA) with an energy-dispersive X-ray analyzer (EDX, Ametek Inc, Berwyn, PA, USA) were employed to analyze both CCEs. AFM measurements were carried out in tapping mode, employing TESPA (NanoWorld, Neuchâtel, Switzerland) probes, featuring a nominal spring constant of 42 N m^−1^ and a resonance frequency of 320 kHz. The AFM images were captured with a scan size of 5 µm × 5 µm in randomly chosen places on the CCE surfaces. AFM topography images were used to determine roughness parameters, including root mean square average roughness (Rq) and surface area difference (SAD), using NanoScope Analysis software (version 1.4, Bruker, Santa Barbara, CA, USA). SEM measurements were performed with an accelerating voltage of 10 kV using a through-the-lens detector (TLD). Elemental surface composition was obtained from EDX spectra, which were acquired through area analysis.

Cyclic voltammetry (CV) measurements were undertaken using an µAutolab type II potentiostat-galvanostat (Eco Chemie, Utrecht, the Netherlands) under the control of GPES software (version 4.9), whereas electrochemical impedance spectroscopy (EIS) experiments were conducted employing an AUTOLAB N128 electrochemical analyzer with FRA2 module (Eco Chemie, Utrecht, the Netherlands) operated by FRA software (version 4.9). Both potentiostats were linked to an M164 electrode stand (MTM Anko Instruments, Cracow, Poland). For these experiments, a three-electrode electrochemical cell configuration was utilized, consisting of Ag|AgCl|3 mol L^−1^ KCl (Mineral, Łomianki-Sadowa, Poland) as the reference electrode, Pt wire (99.99%, The Mint of Poland, Warsaw, Poland) as the counter electrode, and laboratory-made CCEs as the working electrodes.

### 2.4. Electrochemical Procedures

The electrochemical characterization of both CCEs was implemented in 1.0 mol L^−1^ KCl (to assess the potential window width) and 1.0 mmol L^−1^ K_3_[Fe(CN)_6_] solutions (to evaluate the reversibility of the model redox process) using CV and EIS. The cyclic voltammograms in KCl solution were recorded at a scan rate of 100 mV s^−1^, while those of K_3_[Fe(CN)_6_] solution were registered over a scan rate range from 5 to 400 mV s^−1^. The EIS spectra were captured within the frequency ranging from 10,000 to 0.01 Hz (amplitude 10 mV, 50 measuring points).

The electrochemical behavior of PCMC was investigated on the Bi-CCE using CV. Cyclic voltammograms were recorded in BRB at an optimized pH value of 5.0 with a PCMC concentration of 50.0 µmol L^−1^ across the potential range from −0.3 to +1.3 V, employing scan rates ranging from 5 to 400 mV s^−1^. To quantitatively determine PCMC on the Bi-CCE, the SWV was utilized. The PCMC stock solution (1.0 mmol L^−1^) was successively added to the cell containing BRB at pH 5.0, covering concentrations ranging from 0.5 to 58.0 μmol L^−1^. SW voltammograms were recorded with a potential ranging from +0.25 to +1.25 V, using optimized SWV parameters: amplitude of 50 mV, frequency of 60 Hz, and step potential of 5 mV. SWV signals were measured after the baseline correction. Comparative analysis was conducted using the unmodified CCE.

The real sample analysis was conducted in spiked river water samples using the standard addition method. Initially, to study the possible interferences caused by river water components, a blank SW voltammogram was registered for a solution comprising 9.0 mL of BRB at pH 5.0 and 1 mL of unspiked river water. Subsequently, SW voltammogram for the river water sample spiked with PCMC (the cell contained 9.0 mL of BRB at pH 5.0, 1.0 mL of river water spiked with the PCMC stock solution, resulting in a PCMC concentration of 6.0 µmol L^−1^) was recorded. Following this, SW voltammograms were registered for three consecutive additions of the PCMC stock solution (each 60 µL) to the cell containing the river water sample that was spiked with PCMC and diluted with BRB at pH 5.0. SWV signals were measured after the baseline correction.

The effect of interferents (ions) that might be possibly present in river water, i.e., Cd^2+^, Ni^2+^, Cu^2+^, HCO_3_^−^, and SO_4_^2−^, on the PCMC SWV signal was investigated. The ratio of the PCMC concentration to the interferents concentrations in the voltammetric cell was equal to 1:0.5, 1:1, 1:2, 1:10, 1:50, and 1:100.

## 3. Results and Discussion

### 3.1. Surface Characterization of CCEs

**Figure 1:**
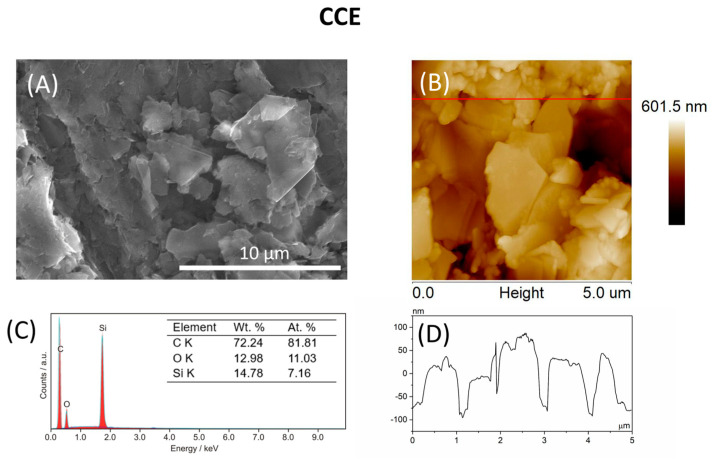

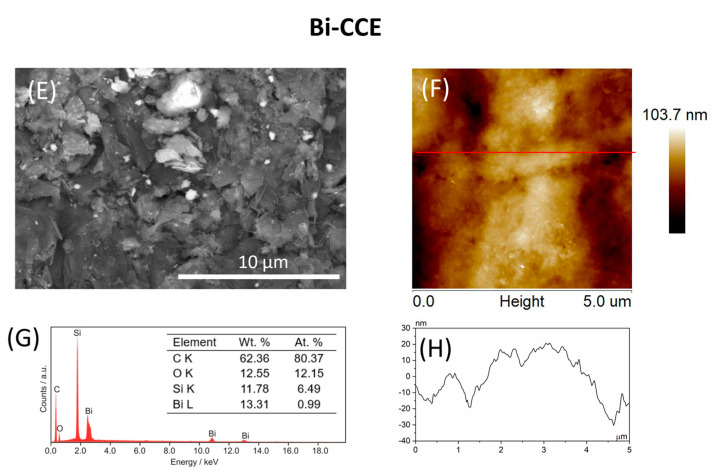
Surface morphology, composition, and topography of the unmodified CCE and Bi-CCE: (**A**,**E**) SEM images, (**C**,**G**) EDX spectra, (**B**,**F**) 2D AFM images, (**D**,**H**) cross-sections. SEM imaging: HV 10 kV, TLD detector, mag. 10,000×. AFM imaging: tapping mode, scan size: 5 μm × 5 μm.

The morphology and topography of both the unmodified CCE and the CCE bulk-modified with the Bi_2_O_3_NPs were examined using SEM and AFM techniques. Both SEM and AFM images of the unmodified CCE, depicted in Figure 1A,B, illustrate flatly arranged graphite flakes covered by a silicon matrix. Elemental analysis shows only C, O, and Si elements, as depicted in Figure 1C. Between individual flakes, noticeable gaps were observed, which is a common feature in such electrodes [12,24,25,27]. In this instance, the observed gaps measure approximately 100 nm deep (Figure 1D). Conversely, SEM and AFM images of the Bi-CCE (Figure 1E,F) show changes in the surface morphology due to the incorporation of Bi_2_O_3_NPs into the ceramic composite. Notably, the morphology of the Bi-CCE differs, presenting a more compact surface with graphite flakes decorated with the Bi_2_O_3_NPs primarily organized in larger agglomerates (Figure 1E). Their presence was confirmed by EDX results indicating ca. 13 wt.% (ca. 0.99 at.%) of Bi_2_O_3_NPs in the ceramic composite (Figure 1G). The AFM results prove a more compact surface of the Bi-CCE (Figure 1F), and the cross-section (Figure 1H) demonstrates that no pinholes are visible. While individual graphite flakes are also distinguished in the AFM image, identifying the Bi_2_O_3_NPs used for modification remains challenging. This difficulty arises from their substantial coverage by the ceramic composite, complicating their distinct visualization in the AFM image. Calculated surface roughness parameter Rq values (88.5 nm for the unmodified CCE, 5.4 nm for the Bi-CCE) indicate reduced roughness after modification. Significant differences in surface morphology are evident upon comparison of the surface area difference (SAD) parameter values between prepared CCEs derived from AFM images shown in Figure 1B,F (16.0% for the unmodified CCE, and 0.5% for the Bi-CCE). These SAD values confirm that the bulk modification of CCE with Bi_2_O_3_NPs leads to a smoother electrode surface with a smaller surface area. Nevertheless, a reduced surface area does not necessarily imply a smaller electroactive surface area, as will be demonstrated by results from subsequent electrochemical measurements. Additionally, from a practical perspective, a smoother electrode surface poses fewer challenges in laboratory applications compared to porous electrodes.

### 3.2. Electrochemical Characterization of CCEs

**Figure 2:**
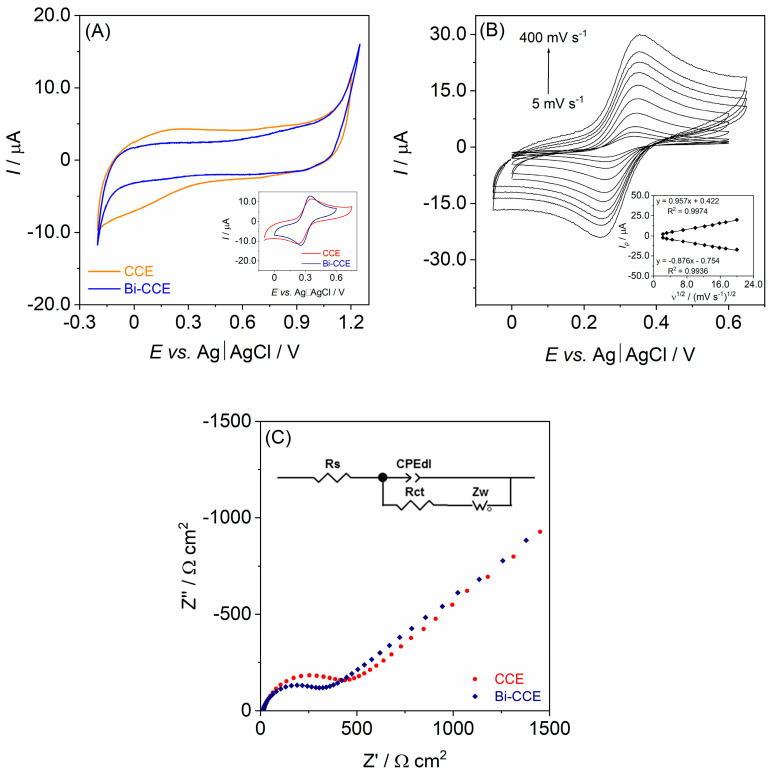
(**A**) Cyclic voltammograms (a scan rate of 100 mV s^−1^) registered in 1.0 mol L^−1^ KCl solution for the unmodified CCE (orange line) and the Bi-CCE (blue line). Inset shows cyclic voltammograms (a scan rate of 100 mV s^−1^) recorded in 1.0 mmol L^−1^ K_3_[Fe(CN)_6_] solution at the unmodified CCE (red line) and the Bi-CCE (navy line); (**B**) Cyclic voltammograms recorded in 1.0 mmol L^−1^ K_3_[Fe(CN)_6_] solution at the Bi-CCE in the scan rates range of 5–400 mV s^−1^. Inset illustrates the relationship between the peaks current (*I_p_*) and the square root of the scan rate (*v*^1/2^); (**C**) EIS spectra captured for 1.0 mmol L^−1^ [Fe(CN)_6_]^4−^/[Fe(CN)_6_]^3−^ in 1.0 mol L^−1^ KCl solution at the unmodified CCE (●) and the Bi-CCE (♦). Frequency range of 10,000–0.01 Hz. Inset exhibits the Randles equivalent circuit consisted of the solution resistance (R_s_), constant phase element describing double-layer capacitance (CPE_dl_), charge transfer resistance (R_ct_), and Warburg impedance related with diffusion (Z_w_).

The initial step in the electrochemical characterization of the prepared CCEs involved determining their working potential window, which is a critical factor indicating the applicability of the working electrodes and providing valuable insights into their surface properties. To assess the potential window width of the Bi-CCE, CV measurements were conducted in a 1.0 mol L^−1^ KCl solution, which is commonly used for characterizing electrode potential windows. The obtained results were then compared to the corresponding curve acquired for the unmodified CCE. As depicted in Figure 2A, the introduction of Bi_2_O_3_NPs into the CCE had an insignificant impact on the potential window width. Both CCEs displayed the same accessible cathodic potential limit (at −0.25 V), while a slightly higher anodic limit was observed in the Bi-CCE (at +1.25 V) compared to the unmodified CCE (at +1.2 V). Significantly, the incorporation of Bi_2_O_3_NPs within the ceramic matrix notably and positively influenced the background current; the Bi-CCE demonstrated a considerably lower capacitive current compared to the unmodified CCE. The heightened background current in the unmodified CCE may be attributed to its more porous surface in contrast to the Bi-CCE. Conversely, the reduced background current observed in the Bi-CCE could be credited to the exceptional properties of Bi_2_O_3_NPs incorporated into the ceramic composite. Moreover, due to the lower background current, it is anticipated that the Bi-CCE will exhibit a lower detection limit than the unmodified CCE.

The subsequent step in the electrochemical characterization of the prepared CCEs involved assessing the reversibility of a model redox marker using CV and the rate of the electron transfer process using EIS. The representative cyclic voltammograms of 1.0 mmol L^−1^ [Fe(CN)_6_]^4−^/[Fe(CN)_6_]^3−^ recorded at a scan rate (*ν*) of 100 mV s^−1^ on both CCEs are depicted in the inset of Figure 2A. While both electrodes exhibited a well-defined redox peak pair, those observed on the Bi-CCE appeared higher than those on the unmodified CCE. Moreover, a peak potential separation (Δ*E_p_*) value closer to the theoretical value of 0.059 V was obtained for the Bi-CCE ([Fe(CN)_6_]^4−^/[Fe(CN)_6_]^3−^ redox probe displayed a Δ*E_p_* value of 0.080 V for the Bi-CCE and 0.113 V for the unmodified CCE), suggesting a more reversible electrode process on the Bi-CCE compared to the unmodified CCE. Furthermore, cyclic voltammograms of the [Fe(CN)_6_]^4−^/[Fe(CN)_6_]^3−^ redox marker were recorded across a scan rate range of 10–400 mV s^−1^ for the Bi-CCE (Figure 2B) as well as for the unmodified CCE (results not shown). In case of both electrodes, a linear dependence of the *I_p_* vs. *v*^1/2^ was observed. Additionally, the *I_a_*/*I_c_* ratio ranged from 0.99 to 1.09 for the Bi-CCE and from 1.03 to 1.12 for the unmodified CCE, closely aligning with the theoretical value of 1. These results indicate the enhanced electron transfer process with improved reversibility of the [Fe(CN)_6_]^4−^/[Fe(CN)_6_]^3−^ redox marker at the Bi-CCE when compared to the unmodified CCE. The EIS spectra (Nyquist plot of real impedance (Z′) vs. imaginary impedance (Z″) fitted by a Randles equivalent circuit) for 1.0 mmol L^−1^ [Fe(CN)_6_]^4−^/[Fe(CN)_6_]^3−^ recorded on both CCEs are depicted in the inset of Figure 2C. The EIS results confirmed a reduction in charge transfer resistance for the Bi-CCE (275.7 Ω cm^2^) when compared to the unmodified CCE (396.3 Ω cm^2^), indicating a more facile redox reaction on this electrode material. All the aforementioned results affirm the beneficial impact of incorporating Bi_2_O_3_NPs into the ceramic composite on the superior electrochemical properties of the modified CCE.

Subsequently, cyclic voltammograms of the [Fe(CN)_6_]^4−^/[Fe(CN)_6_]^3−^ redox marker registered in the ν range of 5–400 mV s^−1^ were utilized to evaluate the effective surface area (*A_eff_*) values for both electrodes. *A_eff_* were determined using the Randles–Sevcik equation [40]: *I_p_* = (2.69 × 10^5^) *n*^3/2^ *A_eff_ D*^1/2^ *v*^1/2^ *c_0_*, where *I_p_* represents peak current (A), *n* denotes the number of transferred electrons (1), *D* signifies the diffusion coefficient of [Fe(CN)_6_]^4−^/[Fe(CN)_6_]^3−^ (7.6 × 10^−6^ cm^2^ s^−1^), *v* denotes the scan rate (V s^−1^), and *c_0_* indicates the redox marker concentration (1.0 × 10^−6^ mol cm^−3^). An increased *A_eff_* value (1.6-times higher) was calculated for the Bi-CCE (*A_eff_* of 4.09 mm^2^) compared to the unmodified CCE (*A_eff_* of 2.59 mm^2^), indicating the advantageous impact of incorporating Bi_2_O_3_NPs in boosting the number of electroactive sites within the ceramic–carbon composite. Furthermore, it is worth noting that the *A_eff_* values smaller than the geometric area value (7.07 mm^2^) were calculated for both CCEs, implying the existence of electrochemically inactive sites on the electrode surface. These electrochemical results confirm the earlier statement that the electroactive surface area is determined not solely by the electrode surface morphology but predominantly by the ratio of electroactive to electrochemically inactive sites on the electrode surface. The incorporation of electroactive Bi_2_O_3_NPs within the electrode material simplifies the process of renewing the electrode surface while preserving its enhanced electroactivity.

### 3.3. Long-Term Stability and Reproducibility Study of the Bi-CCE

**Figure 3:**
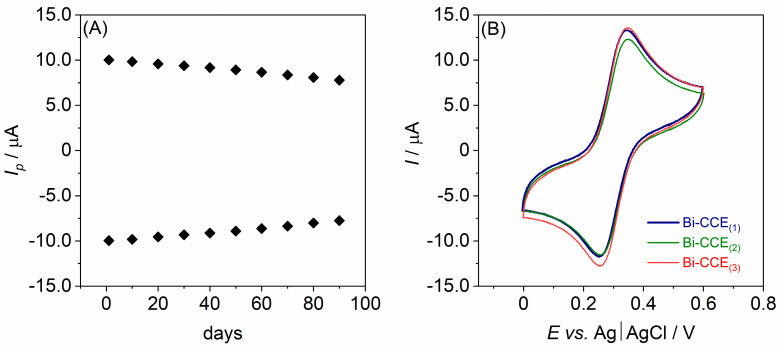
(**A**) The plot representing the changes in the peak current (*I_p_*) over three months (90 days) of the developed Bi-CCE; (**B**) cyclic voltammograms (scan rate of 100 mV s^−1^) in 1.0 mmol L^−1^ K_3_[Fe(CN)_6_] solution at three separate Bi-CCEs.

The long-term stability over time of the developed Bi-CCE was assessed by conducting CV experiments in 1.0 mmol L^−1^ K_3_[Fe(CN)_6_] solution over an extended period. The measurements were systematically performed at regular intervals (every 10 days) for three months (each time involving the renewal of the electrode surface by polishing). The recorded cyclic voltammograms exhibited negligible changes in the *I_p_* values (Figure 3A) indicated by a low relative standard deviation (RSD) value. Specifically, the RSD of *I_p_* remained below 4% for 50 days and below 8% for 90 days. This consistent performance suggests that the Bi-CCE can be considered stable over time and easily renewable through a straightforward polishing procedure.

The reproducibility was assessed by conducting measurements in 1.0 mmol L^−1^ K_3_[Fe(CN)_6_] solution employing three separate Bi-CCEs, each prepared on different days (Figure 3B). RSD lower than 5% was observed, affirming the high reproducibility and reliability of the electrode preparation method.

### 3.4. Electroanalytical Performance of the Bi-CCE

**Figure 4:**
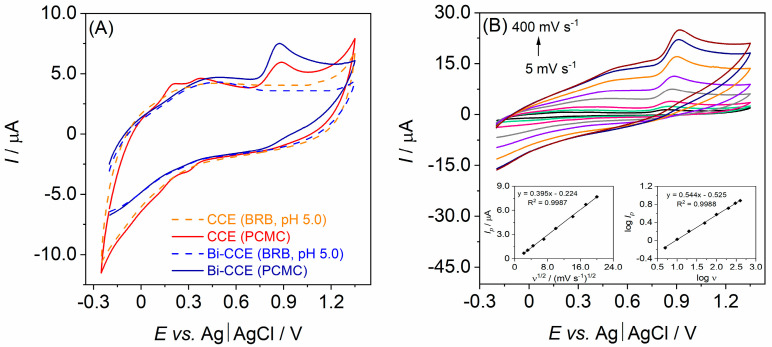
(**A**) Cyclic voltammograms (a scan rate of 50 mV s^−1^) registered in the BRB solution at pH 5.0 (dashed lines) and in the BRB solution containing 50.0 µmol L^−1^ PCMC (solid lines) on the unmodified CCE and the Bi-CCE; (**B**) cyclic voltammograms recorded across scan rates ranging from 5 to 400 mV s^−1^ in the BRB solution at pH 5.0 containing 50.0 µmol L^−1^ PCMC on the Bi-CCE. Insets exhibit the relationship between *I_p_* and *v*^1/2^ (**left graph**) and the dependence of logarithm of *I_p_* vs. logarithm of *v* (**right graph**).

The impact of incorporating Bi_2_O_3_NPs into the CCE on the electroanalytical performance of the Bi-CCE was evaluated using PCMC. Initially, cyclic voltammograms were recorded at a scan rate of 50 mV s^−1^ within the potential range from −0.25 to +1.35 V in the BRB solution at pH 5.0 both in the absence and presence of 50.0 µmol L^−1^ PCMC. As depicted in Figure 4A, PCMC displays a single oxidation peak on both CCEs, which was observed at +0.883 V for the unmodified CCE and +0.865 V for the Bi-CCE. Since no cathodic peak is evident in the cyclic voltammograms when PCMC is present, the electrochemical oxidation of PCMC on both CCEs can be deemed irreversible. Notably, a better-shaped and higher oxidation peak for PCMC (1.6-fold increase in current response) was observed on the Bi-CCE (*I_p_* = 2.43 µA) compared to the unmodified CCE (*I_p_* = 1.52 µA). These initial findings suggest a positive influence resulting from the addition of Bi_2_O_3_NPs on the electroanalytical performance of the CCE in determining PCMC.

The impact of varying scan rates on the electrochemical behavior of PCMC on the Bi-CCE was investigated across the range of 5–400 mV s^−1^. As depicted in Figure 4B, the *I_p_* amplifies with the rising *v*, and the peak potential (*E_p_*) shifts toward more positive values (from +0.84 to +0.92 V). These patterns further affirm the irreversibility of the PCMC oxidation process. Additionally, while a clear linear relationship exists between *I_p_* and *v^1/2^* (R^2^ = 0.9987, as illustrated in the left inset of Figure 4B), the y-intercept deviates from zero, which is contrary to what is expected in a completely diffusion-controlled process where the intercept is typically zero [41]. Moreover, plotting the linear relationship between log *I_p_* and log *v* revealed a slope of 0.544 (R^2^ = 0.9988; displayed in the right inset of Figure 4B), affirming the earlier hypothesis that the electrochemical oxidation process of PCMC is not exclusively controlled by diffusion. Possibly, another mechanism beyond diffusion significantly contributes to the PCMC oxidation process. This observation aligns with typical behaviors observed in phenolic-type compounds [21,42].

Based on the results obtained, it can be stated that the presence of the diffusion-controlled process is beneficial for the analysis being conducted. Thanks to the absence of an adsorption process in the electrooxidation of PCMC, the bulk-modified CCEs were easily reusable after each experiment. To refresh the electrode’s surface, a simple surface-polishing technique was performed only after a series of measurements. In the case of an adsorption process, polishing would be necessary after each scan due to the accumulation of oxidation products on the CCEs surface. On the other hand, an adsorption-controlled process would potentially lead to a decrease in the LOD values in the determination of PCMC. Nevertheless, the presence of the diffusion-controlled process does not limit the electroanalytical application of the sensors and could result in satisfactory validation parameters.

Furthermore, the quantification of PCMC at the Bi-CCE was established through SWV, which is a highly sensitive technique widely employed in electroanalytical applications [8,9]. To ensure an optimally developed PCMC oxidation peak, coupled with a sufficient *I_p_* for precise quantitative analysis, the developed analytical procedure involved optimizing specific conditions. This encompassed determining the ideal pH value of the supporting electrolyte (BRB) and optimizing the SWV parameters such as amplitude, frequency, and step potential.

**Figure 5:**
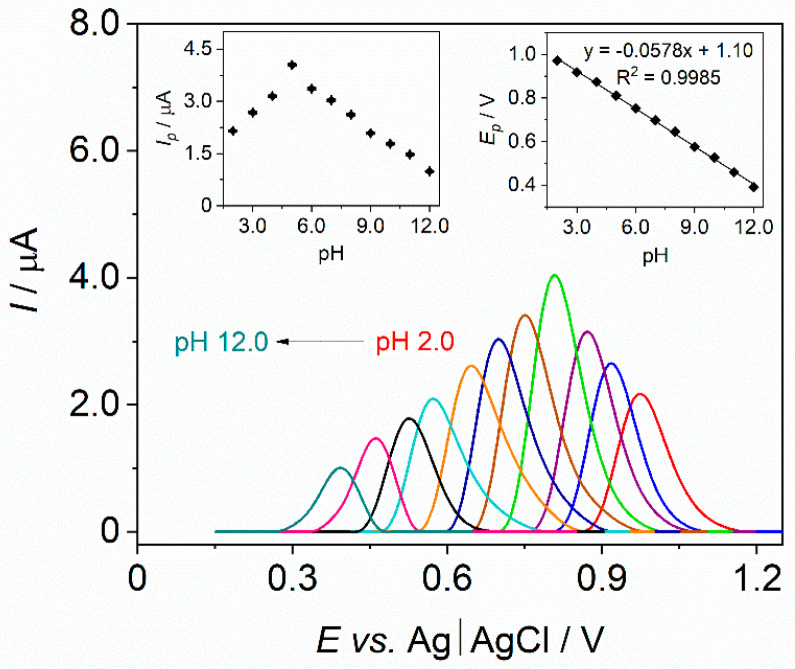
Baseline corrected SW voltammograms of PCMC (50.0 μmol L^−1^) registered on the Bi-CCE in the BRB solutions in the pH range of 2.0–12.0. Insets exhibit the relationship between *I_p_* and pH (**left graph**) with SD error bars (*n* = 3) and the relationship between *E_p_* and pH (**right graph**). SWV parameters: amplitude of 30 mV, frequency of 20 Hz, step potential of 5 mV.

Initially, the impact of pH of the BRB on the current response of PCMC was investigated within a pH range spanning from 2.0 to 12.0 (as depicted in Figure 5). An increase in *I_p_* was noted with rising pH values until reaching 5.0, beyond which a decline in *I_p_* was observed as pH values further increased (as demonstrated in the left inset of Figure 5). The Bi-CCE exhibited the highest PCMC response in the BRB at pH 5.0; thus, this pH value was picked for successive measurements. Furthermore, Figure 5 shows that the *E_p_* of PCMC shifts toward more negative potentials with increasing pH within the 2.0–12.0 pH range, suggesting a pH-dependent oxidation process. Notably, the relationship between *E_p_* and pH is linear (R^2^ = 0.9985; presented in the right inset of Figure 5), with a slope of −0.0578 V pH^−1^, closely resembling the Nernstian value of 0.059 V pH^−1^. This indicates the exchange of an equivalent number of protons and electrons in the electrochemical reaction [43] of PCMC. Based on the obtained results and a literature overview, the electrochemical oxidation mechanism has been proposed, and it can be concluded that one electron and one proton are involved in the electrooxidation of the –OH group present in the PCMC structure [35,36].

Subsequently, the optimization of SWV parameters was conducted across specific ranges: amplitude from 10 to 80 mV, frequency from 10 to 90 Hz, and step potential from 1 to 9 mV. The investigation revealed a gradual increase in the peak current of PCMC with rising SW amplitude, stabilizing around 50 mV. Moreover, the oxidation peak of PCMC broadened notably at amplitudes higher than 50 mV. Across the entire studied SW frequency range, the peak current of PCMC consistently exhibited an increase. Additionally, the investigation highlighted a direct correlation between the increment of step potential value and the increase in the PCMC oxidation peak current. However, the application of step potential values exceeding 5 mV resulted in observable peak distortion. Upon careful evaluation of peak height and shape for PCMC, the optimal experimental conditions were identified as an amplitude of 50 mV, a frequency of 60 Hz, and a step potential of 5 mV.

**Figure 6:**
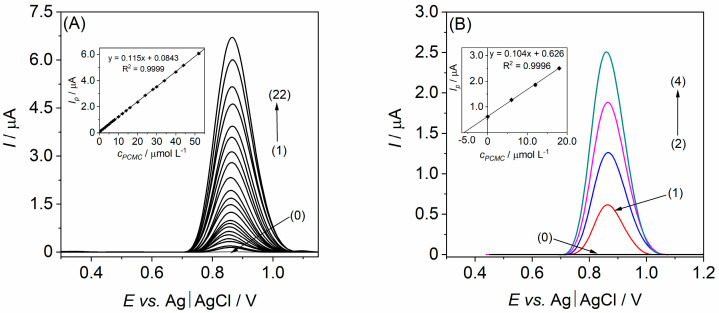
(**A**) Baseline-corrected SW voltammograms recorded on the Bi-CCE in BRB at pH 5.0 containing increasing concentration of the PCMC: (0) blank, (1) 0.5, (2) 1.0, (3) 2.0, (4) 3.0, (5) 4.0, (6) 5.0, (7) 6.0, (8) 7.0, (9) 8.0, (10) 10.0, (11) 12.0, (12) 14.0, (13) 16.0, (14) 20.0, (15) 24.0, (16) 28.0, (17) 30.0, (18) 34.0, (19) 40.0, (20) 44.0, (21) 52.0, and (22) 58.0 µmol L^−1^. SWV parameters: amplitude of 50 mV, frequency of 60 Hz, and step potential of 5 mV. Inset: calibration curve with SD error bars (*n* = 3). (**B**) Baseline-corrected SW voltammograms recorded on the Bi-CCE in the river water sample employing the standard addition method: (0) unspiked river water sample, (1) spiked river water sample, (2) same as (1) + 6.0 µmol L^−1^ PCMC, (3) same as (1) + 12.0 µmol L^−1^ PCMC, (4) same as (1) + 18.0 µmol L^−1^ PCMC. SWV parameters: amplitude of 50 mV, frequency of 60 Hz, and step potential of 5 mV. The inset displays a corresponding standard addition plot with SD error bars (*n* = 3).

Following optimization, the SW voltammograms (Figure 6A) enabled the construction of a calibration curve on the Bi-CCE that facilitated the determination of various essential validation parameters for assessing the developed analytical procedure. To evaluate the impact of the Bi_2_O_3_NPs on the electroanalytical performance of the CCE, similar assessments were conducted on both CCE types, and the resulting validation parameters are detailed in Table 1. The data comparison clearly highlights the beneficial impact of Bi_2_O_3_NPs on the analytical (validation) parameters in the SWV procedure for PCMC determination. The presence of Bi_2_O_3_NPs considerably widened the LDR at the Bi-CCE and extended the linear response limit. Additionally, the Bi-CCE exhibited over an 11-fold increase in sensitivity, which was determined from the slope of the calibration curve, along with notably reduced LOD and LOQ values (nearly 4.5-times lower compared to the unmodified CCE). Furthermore, the Bi-CCE demonstrated improved precision (expressed as RSD of the lowest concentration within the linear range) and accuracy (determined by the recovery of the lowest concentration within the linear range) in comparison with the unmodified CCE. It is noteworthy that both CCEs met the acceptance criteria outlined in the literature [44] for these parameters.

The validation parameters acquired for the Bi-CCE were contrasted with data for various carbon-based electrodes utilized in PCMC determination (Table 2). Overall, the Bi-CCE demonstrates superior electroanalytical performance in terms of both LDR and LOD values compared to most other carbon-based electrodes [24,34,36,37,38]. Remarkably, slightly better, however, still comparable results were achieved using the anodically pretreated boron-doped diamond electrode with a B/C ratio of 2000 ppm [35]. However, it is important to note that the preparation of a boron-doped diamond electrode is an expensive and intricate process that necessitates sophisticated equipment operated by trained personnel. Therefore, this comparison suggests that the developed Bi-CCE in our study exhibits notably low LOD and a wide linear range, indicating its highly efficient performance in PCMC determination.

The efficacy of the optimized and validated SWV procedure for PCMC determination using the Bi-CCE was authenticated through real sample analysis. Initial screening of the samples showed no detectable PCMC. To ascertain its presence, spiking experiments were conducted, and PCMC concentrations were assessed via the standard addition method. Figure 6B displays SW voltammograms derived from successive PCMC additions to the river water sample. The quantification of PCMC content in the river water sample, determined through the linear correlation of *I_p_* vs. PCMC concentration (shown in the inset of Figure 6B), was successfully achieved. The calculated PCMC concentration in the tested sample was 6.21 µmol L^−1^, closely matching the added spiked value of 6.0 µmol L^−1^. Moreover, the RSD values obtained at each concentration level (*n* = 3) did not exceed 3.1%, indicating highly reproducible measurements using the Bi-CCE. The estimated PCMC recovery values ranged from 99.8% to 100.3%, affirming the accuracy of the proposed methodology. Notably, there was no noticeable matrix effect from the analyzed samples on the performance of PCMC. These results affirm the suitability of the Bi-CCE for precise quantitative analysis of PCMC in real samples.

**Figure 7:**
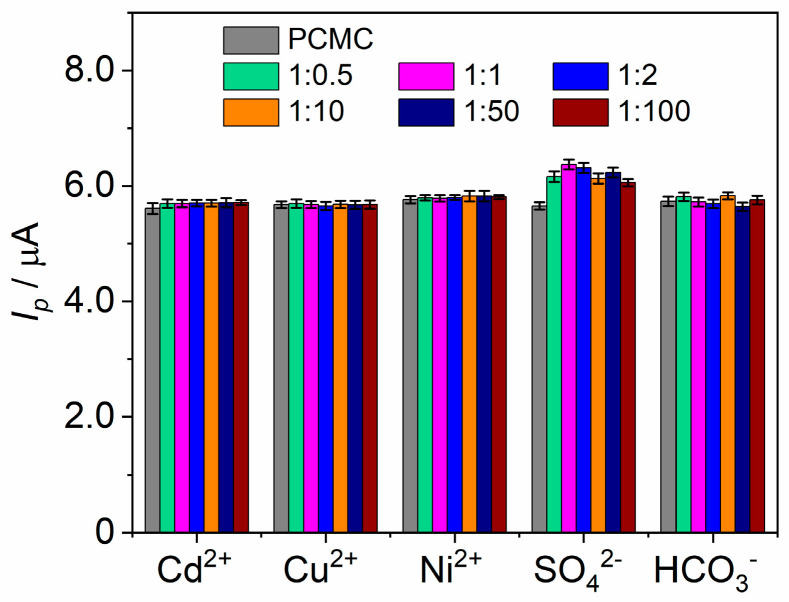
A bar graph depicting the peak current (*I_p_*) of PCMC in the presence of interferents.

Subsequently, to assess method selectivity, various ions commonly present in river water, i.e., Cd^2+^, Ni^2+^, Cu^2+^, HCO_3_^−^, and SO_4_^2−^, were examined (Figure 7). It was observed that the presence of Cd^2+^, Ni^2+^, Cu^2+^, and HCO_3_^−^ ions did not notably interfere with the PCMC signal. However, the presence of SO_4_^2−^ ions resulted in a slight alteration of the PCMC signal. These findings demonstrate a generally favorable selectivity of the developed method.

## 4. Conclusions

In this work, the Bi-CCE was prepared by bulk modification with Bi_2_O_3_NPs and thoroughly investigated using microscopic and electrochemical methods. The results revealed that the incorporation of Bi_2_O_3_NPs into the ceramic matrix significantly altered the morphology and topography of the ceramic composite, resulting in a more compact electrode material when compared to the more porous unmodified CCE, which exhibited visible pinholes. Moreover, the enhancement of the electrochemical properties of the CCE following modification with Bi_2_O_3_NPs was confirmed in the presence of the redox marker. Additionally, the Bi-CCE was successfully verified as an outstanding sensing tool for the reliable, sensitive, and selective determination of the priority environmental pollutant PCMC. Importantly, the exceptional features of the Bi-CCE enabled the development of a direct, simple, and rapid protocol for PCMC determination, demonstrating the possibility of avoiding complex and tedious modification procedures as well as accumulation steps. In summary, all analyses affirm the positive impact of Bi_2_O_3_NPs on the overall performance of the CCE.

As a result, the Bi-CCE developed in this study presents a cheap, prospective, and promising carbon-based electrode material, offering utility as an effective analytical tool for applications in pharmaceutical, clinical, food, and environmental analyses. However, it is important to acknowledge that for the detection of organic compounds in more intricate biological or environmental samples, where concentrations typically fall in the nmol L^–1^ range, additional modifications may be required to further enhance the already superior electroanalytical performance of the Bi-CCE.

## Figures and Tables

**Table 1:** Validation parameters in the SWV procedure of PCMC determination on the unmodified CCE and the Bi-CCE.

Parameter	CCE	Bi-CCE
Linear range (μmol L^−1^)	4.0–24.0	0.5–58.0
Sensitivity (μA L μmol^−1^)	0.0102	0.115
LOD ^(a)^ (μmol L^−1^)	0.73	0.17
LOQ ^(a)^ (μmol L^−1^)	2.21	0.50
Precision ^(b)^ (%)	2.6	0.8
Accuracy ^(b)^ (%)	86.3	101.2

^(a)^ LOD = (3.3 × SD_a_)/b; LOQ = (10 × SD_a_)/b, where SD_a_ is the standard deviation of the slope, b is the intercept value; ^(b)^ precision and accuracy calculated for 3 consecutive measurements for PCMC concentration of 4.0 µmol L^−1^ at the unmodified CCE and 0.5 µmol L^−1^ at the Bi-CCE.

**Table 2:** Comparison of PCMC determination on various carbon-based electrodes.

Electrode	Linear Range (µmol L^−1^)	LOD (µmol L^−1^)	Ref.
GCE	21.0–210.4	9.3	[37]
MWCNTs-GCE	14.0–137.5	8.8	[34]
UiO-66-NH2@PEDOT/GA-GCE	0.6–18.0	0.20	[36]
APT-BDDE (B/C = 2000 ppm)	0.5–100.0	0.11	[35]
SNG–C–PANI	0.7–7.0	0.69	[38]
MWCNTs-CCE	3.0–32.0	0.71	[24]
Bi-CCE	0.5–58.0	0.17	This work

GCE—glassy carbon electrode; MWCNTs-GCE—glassy carbon electrode modified with multi-walled carbon nanotubes; APT-BDDE—anodically pretreated boron-doped diamond electrode; MWCNTs-CCE—carbon ceramic electrode modified with multi-walled carbon nanotubes; UiO-66-NH_2_@PEDOT/GA-GCE—glassy carbon electrode modified with Zr-based metal–organic framework (UiO-66-NH_2_), poly-(3,4-ethylenedioxythiophene) and graphene aerogel; SNG–C–PANI—sonogel carbon poly-aniline electrode; Bi-CCE—carbon ceramic electrode bulk-modified with Bi_2_O_3_ nanoparticles.

## Data Availability

Data will be made available upon request.
